# Relationship between the cross-sectional area of the lumbar dural sac and lower urinary tract symptoms: A population-based cross-sectional study

**DOI:** 10.1371/journal.pone.0271479

**Published:** 2022-08-11

**Authors:** Tetsushi Oyama, Kanichiro Wada, Kazushige Koyama, Gentaro Kumagai, Sunao Tanaka, Toru Asari, Atsushi Imai, Teppei Okamoto, Shingo Hatakeyama, Songee Jung, Yoshikuni Sugimura, Chikara Ohyama, Yasuyuki Ishibashi

**Affiliations:** 1 Department of Orthopaedic Surgery, Hirosaki University Graduate School of Medicine, Hirosaki, Aomori, Japan; 2 Department of Urology, Hirosaki University Graduate School of Medicine, Hirosaki, Aomori, Japan; 3 Department of Digital Nutrition and Health Sciences, Hirosaki University Graduate School of Medicine, Hirosaki, Aomori, Japan; 4 Department of Microbial Flora and Health Science, Hirosaki University Graduate School of Medicine, Hirosaki, Aomori, Japan; Saint George’s Hospital Medical School: St George’s University of London, IRAQ

## Abstract

This study aimed to investigate the relationship between the cross-sectional area of the dural sac (DCSA) and lower urinary tract symptoms (LUTS). This study included 270 Japanese participants from a community health check-up in 2016. Overactive bladder (OAB) was diagnosed during the assessment of LUTS. The smallest DCSA of each participant was defined as the minimum DCSA (mDCSA). The cutoff size of the mDCSA in OAB was evaluated using receiver operating characteristic analysis. Multiple logistic regression analyses were performed to identify the independent risk factors for OAB, and a scoring system was developed for estimating these. The prevalence of OAB was 11.1%. Age and low back pain visual analogue scale (LBP VAS) scores were significantly higher, and the mean mDCSA was significantly lower in participants with OAB than in those without. The cutoff size of mDCSA in OAB was 69 mm^2^. There were significant correlations between OAB and age, LBP VAS score, and mDCSA<70 mm^2^. Lumbar spinal stenosis (LSS) should be considered a cause of LUTS when mDCSA is <69 mm^2^. Assessing the mDCSA with age and LBP VAS score was more valuable in detecting LUTS in LSS than the mDCSA alone.

## Introduction

Lumbar spinal stenosis (LSS) refers to the narrowing of the spinal canal, leading to compression of the cauda equina and nerve roots; this compression results in symptoms such as low back pain (LBP), leg pain/numbness, intermittent claudication (IC), and lower urinary tract symptoms (LUTS) [1–3]. LSS is diagnosed based on patient history, physical examination results, and magnetic resonance imaging (MRI) findings. Patients with symptomatic LSS generally demonstrate low quality of life (QOL) scores [1]. LUTS in LSS shows both storage and voiding symptoms [4]. LUTS was also reported to decrease QOL in several population studies [5, 6]. Age, obesity, and vascular disorders have been postulated as risk factors [7]; therefore, it is important to prevent LUTS through early detection and appropriate intervention.

Some studies have demonstrated a correlation between LUTS and the degree of stenotic compression of the dural sac in patients with LSS treated either surgically or conservatively [8, 9]. Plain radiographs or computed tomography (CT) was used in some previous reports [10, 11], and the anteroposterior diameter (APD) of the dural sac was used to evaluate LSS [8, 10, 11].

Bladder dysfunction is often difficult to diagnose, as some cases are asymptomatic, and it may develop through LSS as well as the urinary tract itself. Prior studies have used the cross-sectional area of the dural sac (DCSA) on MRI in healthy volunteers to report the presence of radiographically asymptomatic LSS [12]; however, it remains unclear how the DCSA on MRI is related to LUTS in the Japanese population. In addition, there is little knowledge regarding biomarkers other than LSS related to LUTS that can be used for early detection.

Further investigation regarding the association between multifactorial parameters, including LUTS and the DCSA, is needed to clarify this point. This study aimed to estimate the prevalence of LUTS in the Japanese population and evaluate the relationship between radiographical, physiological, and chemical parameters and LUTS.

## Materials and methods

### Participants and study design

This study was approved by the Ethics Committee of our institution; all participants provided written informed consent (Number; 2016–028, Approval date; May 27, 2016). Study data were derived from a community-based public health project introduced by our institution in 2005, aiming to help the general population maintain a longer lifespan. The project provides annual health check-ups for approximately 1000 volunteers who live in a city in northern Japan and are at least 20 years old; it collects basic anthropometric and lifestyle data, as well as biomechanical data, biochemical blood and urine test results, questionnaire results, and test findings related to examinations by various specialists, with 1115 participants in 2016. Scores pertaining to lifestyle and medical history were retrieved, and the participants’ blood pressure (systolic blood pressure [SBP], diastolic blood pressure [DBP], and ankle-brachial index), body weight, and height were measured. Blood samples were collected through venipuncture.

The intensities of LBP, leg pain, and leg numbness were measured using the visual analogue scale (VAS). Lumbar spine radiography and MRI were randomly conducted for 290 participants. Of these, ten were excluded (two with a prior history of OAB, four with benign prostatic hyperplasia. and four with missing data who did not answer the questionnaire entirely). Finally, 129 men and 141 women between 24 and 85 years of age were analysed (average age at enrolment: men, 53.3±14.9 years; women, 54.3±14.6 years).

### Lifestyle, medical history, and blood data

We collected data pertaining to their daily smoking habits (0, never smoked; 1, current or ex-smoker), alcohol consumption (0, ex-drinker, never drank, or social drinker; 1, habitual drinker), and sleeping time (min/day), and medical history (history of hypertension [HT], dyslipidaemia [DL], diabetes mellitus [DM] and osteoporosis). Blood samples were collected to measure high-density lipoprotein cholesterol, triglyceride (TG), haemoglobin A1c (HbA1c), fasting glucose (FGS), creatinine, and bone metabolism markers (total type 1 procollagen N-terminal propeptide [NTX], bone tarte-resistant acid phosphate-5b, pentosidine, and homocysteine).

### Evaluation of LUTS

LUTS was evaluated using the overactive bladder symptom score (OABSS), a measure of assessing urinary urgency validated by Homma [5]. The OABSS is a self-administered, self-reported history questionnaire for diagnosing OAB and is the sum score of symptoms: daytime frequency (Q1), night-time frequency (Q2), urgency (Q3), and urgency incontinence (Q4). Participants were diagnosed with OAB if their Q3 score was ≥2 points, and the total score was ≥3 points.

### Radiography of the lumbar spine

Each participant underwent lateral lumbar spine radiography in a neutral standing position. The Kellgren-Lawrence grading system (K-L grade) was used to evaluate disc degeneration as follows: grade 0, normal disc with no osteophytes; grade 1, slight anterior wear and osteophyte formation; grade 2, definite anterior wear and mild disc space narrowing, with osteophyte formation; grade 3, moderate disc space narrowing with osteophytes and endplate sclerosis; and grade 4, large osteophytes, marked disc space narrowing, and endplate sclerosis [13]. The K-L grade was measured at the L1/L2 to L5/S1 disc level and was assigned by an orthopaedic surgeon. The most severe level was chosen as the representative grade, and the interobserver variability for measuring the K-L grade was confirmed by the intraclass correlation coefficient, determined to be 0.878.

### MRI of the lumbar spine

A mobile MRI unit (1.5T Intera, Phillips, Amsterdam, the Netherlands) was used in our study, and each participant underwent a lumbar spinal MRI. The exclusion criteria were the presence of cardiac pacemakers, claustrophobia, or other contraindications. Participants were placed in the supine position with their legs straight during MRI; the protocol included sagittal (repetition time, 4000 ms/echo; echo time, 120 ms; field of view, 180×180 mm) and axial (repetition time, 4000 ms/echo; echo time, 120 ms; field of view, 320×320 mm) T2-weighted fast spin echo imaging.

### Evaluation of the DCSA and morphology on MRI of the lumbar spine

**Fig 1:**
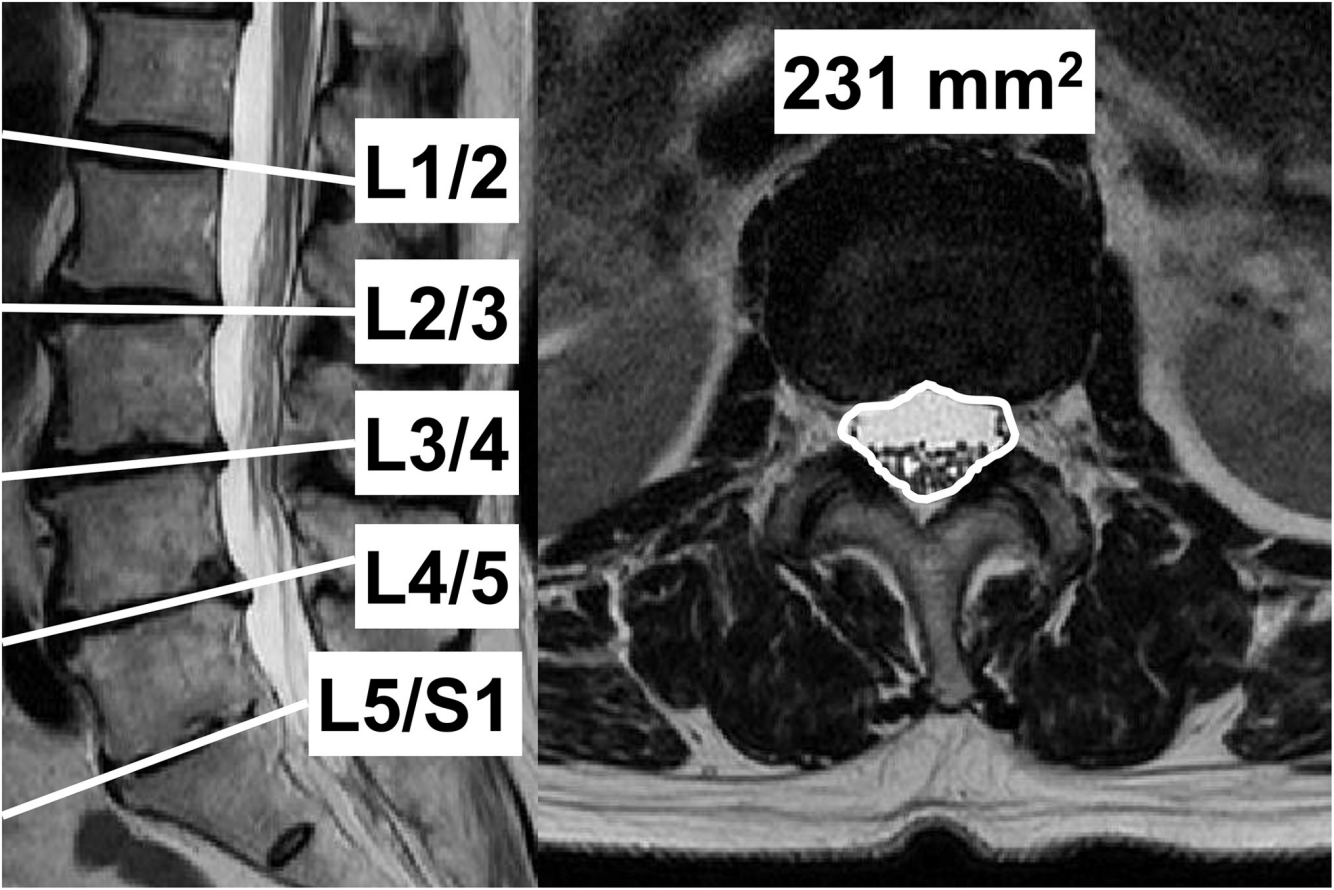
Measurements of the DCSA using MRI. Sagittal (left) and axial (right) view of the L1/2 disc level of a participant. The DCSA is surrounded by a white dotted line using axial MRI at each disc level (straight white line).

The DCSA was measured at the L1/L2 to L5/S1 disc level; axial images were displayed and analysed using ImageJ digital image viewing software (National Institutes of Health, Bethesda, MD, USA). The circumference of each dural sac was outlined using a graphic cursor, and DCSAs were identified by manual tracing in mm^2^ (Fig 1). The DCSA was measured by an orthopaedic surgeon; interobserver variability for measuring the DCSA was confirmed by the intraclass correlation coefficient, determined to be 0.933. After measuring the DCSA, we defined the smallest DCSA of each participant as the minimum DCSA (mDCSA).

We also evaluated the morphologic classification of the dural sac at the mDCSA level described in a previous report [14]. The classification is based on cerebrospinal fluid (CRF) and rootlet content as follows, Grade A stenosis: there is clearly CSF visible inside the dural sac, but its distribution is inhomogeneous. Grade B stenosis: the rootlets occupy the whole of the dural sac, but they can still be individualized. Some CSF is still present giving a grainy appearance to the sac. Grade C stenosis: no rootlets can be recognized, the dural sac demonstrating a homogeneous gray signal with no CSF signal visible. There is epidural fat present posteriorly. Grade D stenosis: in addition to no rootlets being recognizable there is no epidural fat posteriorly. The morphologic classification was also evaluated by an orthopaedic surgeon; interobserver variability for measuring the morphologic classification was confirmed by the intraclass correlation coefficient, determined to be 0.873.

### Statistical analysis

Participants were divided into the OAB+ and OAB- groups. The demographic data were compared between the two groups using the Mann-Whitney U or chi-square test. We also investigated the mDCSA level to evaluate the most frequent disc level and compared the two groups using the chi-square test. The cutoff size of the mDCSA was evaluated using receiver operating characteristic (ROC) analysis, and the OABSS was compared between the four groups (mDCSA <70 mm^2^, 70 mm^2^≤mDCSA<110 mm^2^, 110 mm^2^ ≤mDCSA<150 mm^2^, 150 mm^2^≤mDCSA) using the Tukey honest significant difference test. Spearman’s rank correlation was used to assess the correlations between OAB, age, and mDCSA. To evaluate the multi-variable relationship between OAB and the mDCSA, we conducted a single logistic regression analysis with the demographic data as independent variables and the prevalence of OAB as the dependent variable. Based on this analysis, all variables associated with the prevalence of OAB with P<0.10 in the single logistic analysis were included in multiple logistic analysis. Next, we conducted stepwise multiple logistic regression analysis with the aforementioned variables as independent variables and the prevalence of OAB as the dependent variable.

Finally, we derived a scoring system rounding each odds ratio (OR) of the stepwise multiple logistic analysis to the nearest integer (Table 1); then, the integer values from all applicable factors were added together to estimate each participant’s total score (Table 2). We used the ROC analysis results to evaluate the cutoff value for OAB. All analyses were performed using the statistical program IBM SPSS Statistics 26.0 (IBM Corp., Chicago, IL, USA). Statistical significance was set at P<0.05, except in the single logistic analysis (P<0.10).

**Table 1:** Demographic data between the OAB+ group and OAB- group.

	OAB+ (n = 30)	OAB- (n = 240)	P-value
Age (years)*	64.6 ± 11.5	52.5 ± 14.5	<0.001
Sex (male: female), n	16 : 14	113 : 127	0.519
BMI (kg/m^2^)	24.1 ± 3.18	23.2 ± 3.06	0.147
Lifestyle and medical history			
Alcohol user, n (%)	15 (50)	120 (50)	0.577
Smoker, n (%)	3 (10)	44 (18)	0.192
Sleeping time (minutes)	440 ± 86.0	417 ± 64.0	0.251
HT, n (%)*	15 (50)	52 (22)	0.001
DL, n (%)*	8 (27)	32 (13)	0.053
DM, n (%)	4 (13)	18 (8)	0.272
Osteoporosis, n (%)	1 (3)	5 (2)	0.501
VAS (mm)			
LBP*	28.8 ± 24.4	17.9 ± 21.3	0.012
Leg pain	7.17 ± 16.7	5.02 ± 13.4	0.560
Leg numbness	6.13 ± 12.6	4.81 ± 13.0	0.697
Kellgren-Lawrence grade (lumbar)			
Grade 0, n (%)*	2 (7)	88 (37)	0.007
Grade 1, n (%)	9 (30)	72 (30)	0.473
Grade 2, n (%)*	13 (43)	54 (23)	0.015
Grade 3, n (%)	5 (17)	21 (9)	0.145
Grade 4, n (%)	1 (3)	5 (2)	0.510
mDCSA (mm^2^)*	99.53 ± 47.0	118.13 ± 43.2	0.024
Morphologic classification			
Grade A, n (%)*	18 (60)	216 (90)	<0.001
Grade B, n (%)*	7 (23)	11 (5)	0.001
Grade C, n (%)*	5 (17)	13 (5)	0.036
Grade D, n (%)	0 (0)	0 (0)	-
SBP (mmHg)*	131.2 ± 18.6	123.5 ± 17.2	0.033
DBP (mmHg)	77.3 ± 10.7	75.7 ± 11.3	0.280
Rt. ABI	1.13 ± 0.07	1.14 ± 0.08	0.607
Lt. ABI	1.13 ± 0.06	1.13 ± 0.07	0.926
HDL-C (mg/dL)	61.1 ± 14.6	64.9 ± 18.3	0.258
TG (mg/dL)*	117.7 ± 68.4	96.7 ± 56.2	0.043
HbA1c (%)*	6.2 ± 1.0	5.8 ± 0.6	0.003
FGS (mg/dL)*	102.4 ± 26.9	90.8 ± 14.9	0.006
Cr (mg/dL)	0.73 ± 0.11	0.74 ± 0.21	0.850
Bone metabolism markers			
P1NP (μg/L)*	42.1 ± 18.7	46.1 ± 17.4	0.051
NTX (nmol BCE/L)	14.5 ± 3.7	15.1 ± 3.6	0.342
TRACP-5b (mU/dL)	399.1 ± 159.1	410.4 ± 167.0	0.633
Pentosidine (pmol/mL)*	35.6 ± 20.9	26.8 ± 9.7	0.008
Homocysteine (nmol/mL)	8.8 ± 2.4	9.6 ± 3.7	0.549

Values are the median±standard deviation or n (%)

BMI, body mass index; Rt, right; Lt, left; ABI, ankle-brachial index; HDL-C, high density lipoprotein cholesterol; FGS, fasting glucose; Cr, creatinine; P1NP, total type 1 procollagen N-terminal propeptide; TRACP-5b; bone tarte-resistant acid phosphate-5b

*p<0.050

**Table 2:** Disc level of the mDCSA.

Disc level	OAB+ (n = 30)	OAB- (n = 240)	P-value
L1/L2	1 (3)	8 (3)	1.000
L2/L3	4 (13)	23 (10)	0.518
L3/L4	9 (30)	48 (20)	0.206
L4/L5	11 (37)	69 (29)	0.371
L5/S1*	5 (17)	92 (38)	0.020

Values are n (%).

*p<0.050

## Results

### Comparison of backgrounds, blood data, lumbar spinal symptoms, and MRI findings between the OAB+ and OAB- groups

Table 3 shows participants’ characteristics, including demographic measurements between the OAB+ and OAB- groups; the prevalence of OAB was 11.1% (n = 30). Age was significantly higher (64.6 vs 52.5 years; P<0.01) and the mean mDCSA was significantly smaller (99.5 mm^2^ vs 118.13 mm^2^; P = 0.024) in the OAB+ group than in the OAB- group. There was also a significant between-group difference in the low back pain visual analogue scale (LBP VAS) score, history of HT, SBP, and levels of TG, HbA1c, FGS, and pentosidine (Table 3).

**Table 3:** Results of the single logistic analysis of the factors that correlated with the prevalence of OAB.

Independent variable	B	OR	95% CI	P-value
Age (20–49, 50–59, 60–69, ≥70 years)*	0.538	1.713	1.288–2.278	<0.001
Sex	-0.250	0.779	0.364–1.666	0.519
BMI	0.096	1.100	0.979–1.237	0.108
Alcohol user	0.000	1.021	0.468–2.136	1.000
Smoker	-0.703	0.495	0.114–1.705	0.265
Sleeping time*	0.005	1.005-	1.000–1.011	0.073
HT*	1.285	3.615	1.659–7.877	0.001
DL*	0.860	2.364	0.970–5.760	0.058
DM	0.641	1.897	0.597–6.035	0.278
Osteoporosis	0.483	1.621	0.183–14.357	0.664
LBP VAS score (25≤VAS score, 25<VAS score≤50, 50<VAS score ≤75, 75<VAS score)*	0.580	1.787	1.142–2.796	0.011
Leg pain VAS score (25≤VAS score, 25<VAS score≤50, 50<VAS score ≤75, 75<VAS score)	0.198	1.218	0.539–2.753	0.635
Leg numbness VAS score (25≤VAS score, 25<VAS score≤50, 50<VAS score≤75, 75<VAS score)	0.059	1.061	0.394–2.857	0.907
K-L grade (0,1,2,3,4)*	0.567	1.764	1.232–2.524	0.002
mDCSA<70 (mm^2^)*	1.466	4.333	1.909–9.837	<0.001
mDCSA level (L3/4 or L4/5)*	0.743	0.069	0.945–4.680	<0.001
Morphologic classification (A, B, C, D)*	0.939	2.558	1.533–4.268	<0.001
SBP (mmHg)*	0.024	1.024	1.003–1.046	0.026
DBP (mmHg)	0.012	1.012	0.979–1.047	0.470
Rt. ABI	-0.014	0.986	0.939–1.035	0.573
Lt. ABI	0.000	1.000	0.951–1.052	0.986
HDL-C	-0.013	0.987	0.965–1.011	0.283
TG*	0.005	1.005	1.000–1.011	0.066
HbA1c*	0.778	2.178	1.280–3.706	0.004
FGS*	0.028	1.028	1.011–1.046	0.002
Cr	-0.378	0.685	0.078–6.019	0.773
P1NP	-0.014	0.986	0.962–1.010	0.242
NTX	-0.042	0.959	0.859–1.071	0.457
TRACP-5b	0.000	1.000	0.997–1.002	0.724
Pentosidine*	0.047	1.048	1.017–1.079	0.002
Homocysteine	-0.074	0.928	0.816–1.056	0.259

B: regression coefficient; 95% CI: 95% confidence interval of the odds ratio.

Dependent variable: OAB.

BMI, body mass index; FGS, fasting glucose; Cr, creatinine; P1NP, total type 1 procollagen N-terminal propeptide; TRACP-5b, bone tarte-resistant acid phosphate-5b

*p<0.100

The most frequently observed mDCSA disc levels were L4/5 (40.0%), followed by L3/4 (33.3%) and L5/S1 (16.7%) in the OAB+ group; and L5/S1 (38.3%), L3/4 (30.4%), and L4/5 (20.0%) in the OAB- group (Table 4). However, there was significant difference only at the L5/S1 level between the two groups.

**Table 4:** Results of stepwise multiple logistic regression analyses relative to OAB.

	B	OR	95% CI	P-value
Age (20–49, 50–59, 60–69, ≥70 years)	0.459	1.582	1.177–2.127	0.002
LBP VAS score (25≤VAS score, 25<VAS score≤50, 50<VAS score≤75, 75<VAS score)	0.590	1.805	1.102–2.955	0.019
mDCSA<70 mm^2^	1.182	3.261	1.357–7.835	0.008

B: regression coefficient; 95% CI: 95% confidence interval of the odds ratio.

Dependent variable: OAB; independent variables: Age, sleeping time, HT, DL, LBP VAS score K-L grade, mDCSA<70 mm^2^, mDCSA level, morphologic classification, SBP, TG, HbA1c, FGS, and pentosidine.

### OABSS between the four groups by the mDCSA

**Fig 2:**
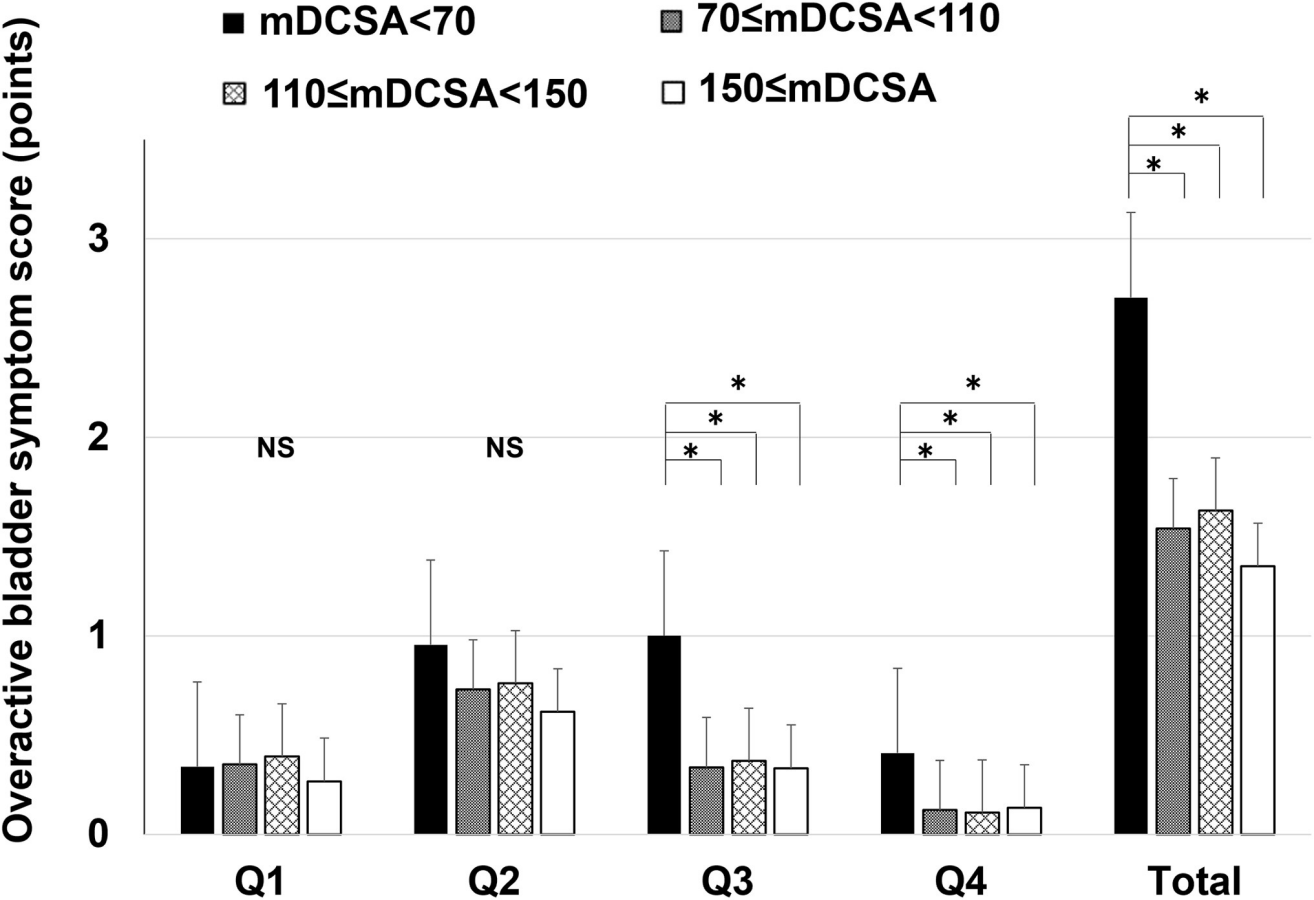
OABSS and total score of each question. Daytime frequency, Q1; night-time frequency, Q2; urgency, Q3; urgency incontinence, Q4. The bars represent the groups: mDCSA<70 mm2, 70 mm2≤mDCSA<110 mm2, 110 mm2≤mDCSA<150 mm2, and 150 mm2≤mDCSA, from left to right. * indicates a significant between-group difference (P<0.05); NS indicates no significant difference.

The OABSS results are summarised in Fig 2. The scores for Q3, Q4, and the total score were significantly higher in the mDCSA<70 mm^2^ group than in the other groups, while Q1 and Q2 scores showed no significant between-group differences (Fig 2).

### Relationship between the OABSS and mDCSA

**Fig 3:**
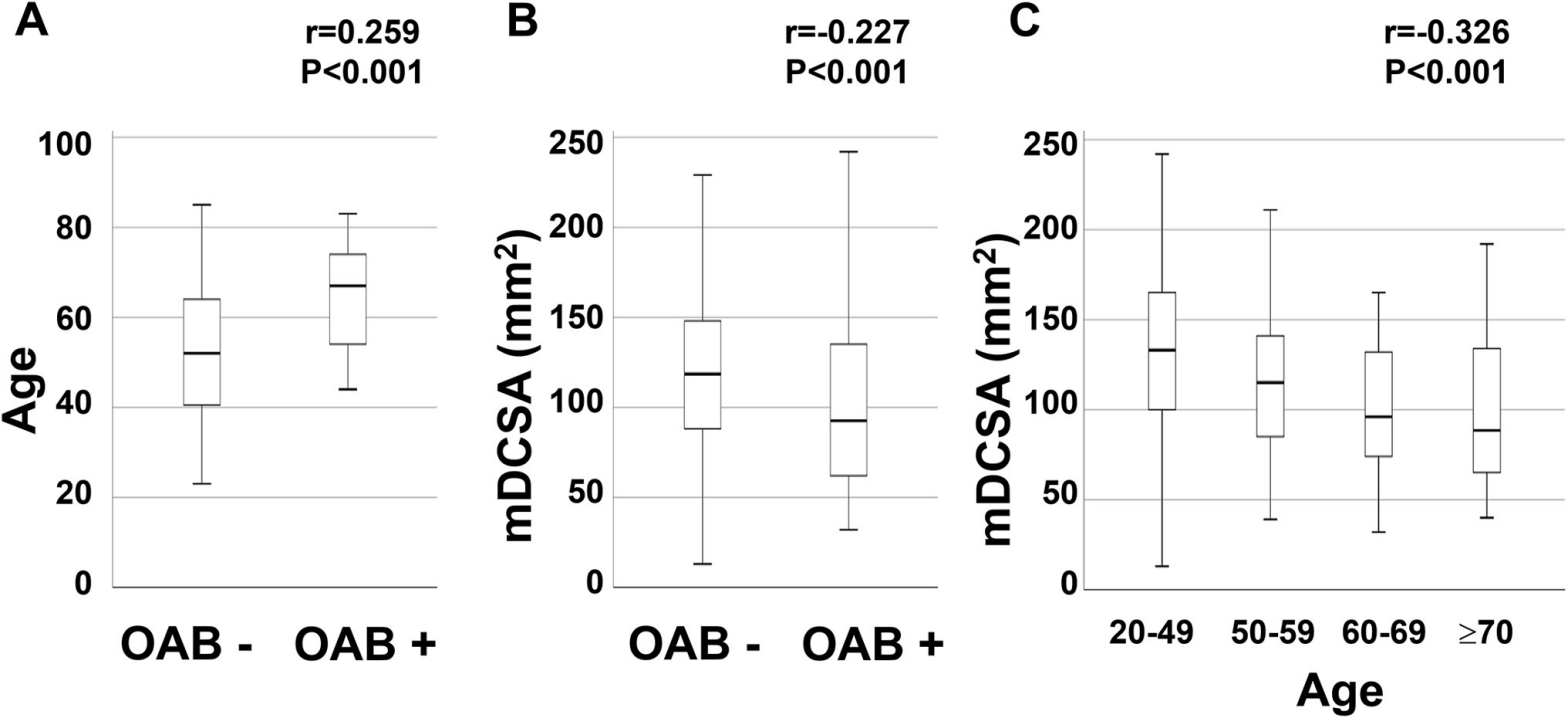
Box plot of OAB, age, and mDCSA. Box plot of OAB/ age (A), OAB/ mDCSA (B), age/ mDCSA. Each number above the box plot represents the result of Spearman’s rank correlation (r: correlation coefficient, P: statistical significance).

**Fig 4:**
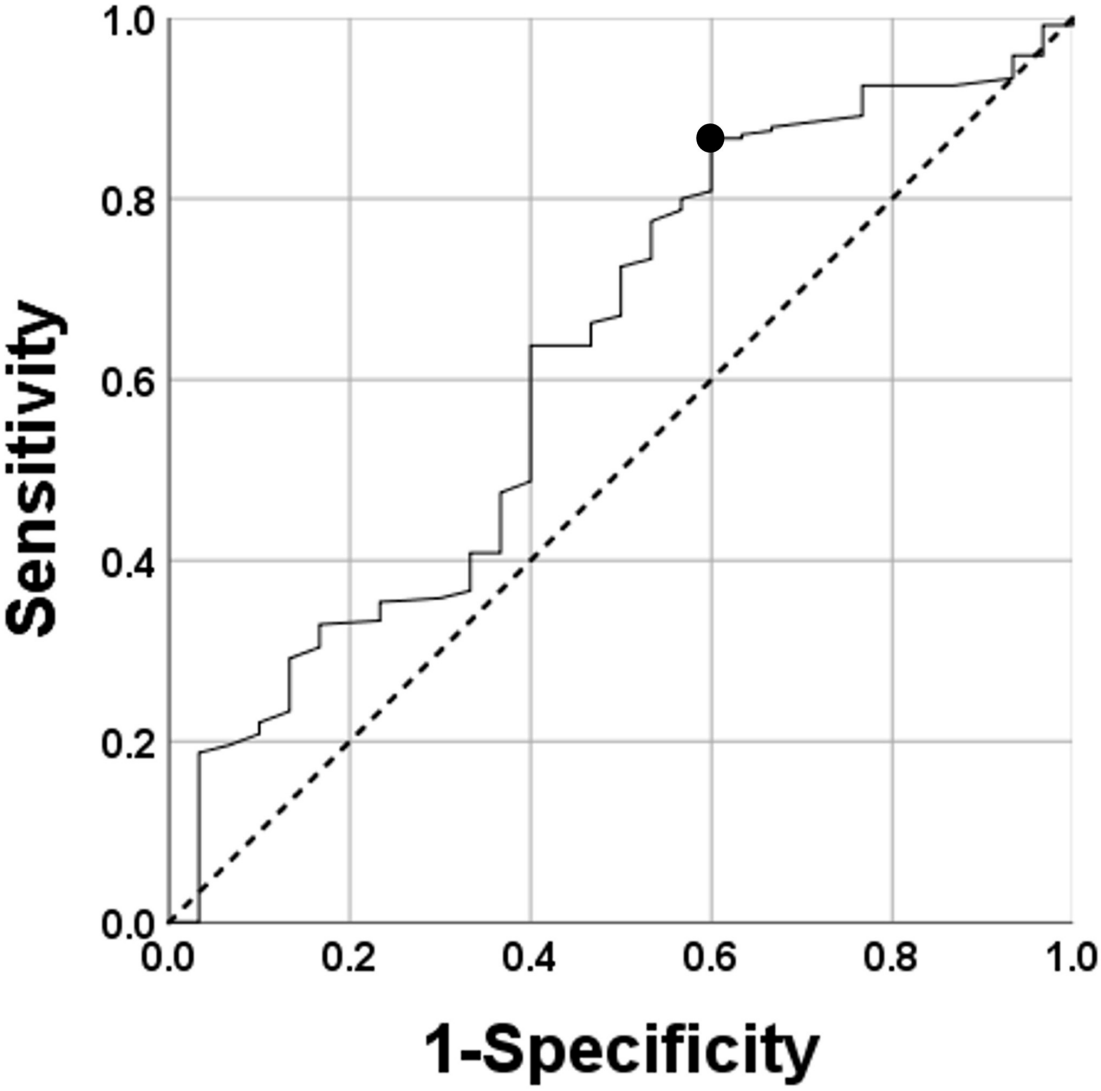
Cutoff mDCSA. The solid black line represents the ROC curve for OAB to define the cutoff mDCSA. The dotted line represents the diagonal reference line. The cutoff mDCSA, calculated from this curve, was 69 mm2 (sensitivity: 86.7%; specificity: 40.0%). The large black dot indicates the point at which the cutoff was calculated.

Results of Spearman’s rank correlation OAB are summarised in Fig 3. OAB was positively correlated with age (r = 0.259, P<0.001) and negatively correlated with mDCSA (r = − 0.227, P<0.001). mDCSA was negatively correlated with age (r = -0.326, P<0.001) (Fig 3). The ROC analysis evaluating the cutoff size of the mDCSA is summarised in Fig 4. The cutoff size of the mDCSA for OAB was 69 mm^2^ (sensitivity, 86.7%; specificity, 40.0%), while the area under the curve (AUC) was 0.626 (Fig 4). Results of single logistic regression analyses are summarised in Table 5. Age (categorised into 20–49, 50–59, 60–69, and ≥70 years), sleeping time, HT, DL, LBP VAS score (categorised into VAS score ≤25, 25<VAS score≤50, 50<VAS score≤75, and VAS score>75), Kellgren-Lawrence grading system (K-L) grade, mDCSA<70 mm^2^, mDCSA level, morphologic classification, SBP, and levels of TG, HbA1c, FGS, and pentosidine were correlated.

**Table 5:** Scoring system based on significant independent factors of multiple logistic regression analyses.

Age (years)	20–49	50–59	60–69	≥70
Points	0	2	4	6
Low back pain VAS score	25≤ VAS score	25< VAS score ≤50	50< VAS score ≤75	VAS score >75
Points	0	2	4	6
mDCSA	≥70 mm^2^	<70 mm^2^		
Points	0	3		

Results of the stepwise multiple logistic regression analyses are summarised in Table 1. Since there was no collinearity (collinearity when |r|>0.80) between mDCSA<70mm^2^ and morphologic classification (Spearman’s rank correlation r = -0.478, P<0.001), we added these as independent variables in the analyses. There were significant correlations between OAB and age (regression coefficient [B]: 0.057; 95% confidence interval [CI]: 1.025–1.092; OR: 1.058; P<0.001), LBP VAS score (B: 0.021; 95% CI: 1.004–1.039; OR: 1.021; P< 0.016), and mDCSA<70 mm^2^ (B: 1.114; 95% CI: 1.289–7.197; OR: 3.221; Table 1).

**Fig 5:**
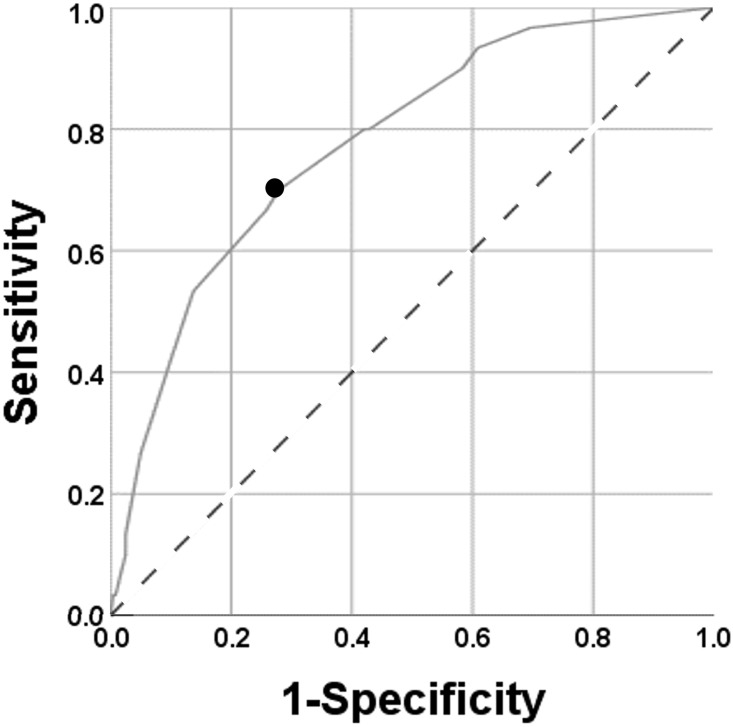
Cutoff value of the scoring system. The solid black line represents the ROC curve for OAB to define the cutoff value of the scoring system. The dotted line represents the diagonal reference line. The cutoff value of the scoring system, calculated from this curve, was 7.5 points (sensitivity: 70.0%; specificity: 72.1%). The large black dot indicates the point at which the cutoff value was calculated.

Results of the final ROC analysis are summarised in Fig 5. The cutoff value of the scoring system for OAB was 7.5 points (sensitivity, 70.0%; specificity, 72.1%), and the AUC was 0.721 (Fig 5).

## Discussion

The results of this study revealed the prevalence of LUTS in a community-dwelling Japanese population to be 11.1%, with a significant between-group difference regarding the mDCSA (the cutoff size of which was 69 mm^2^). This is the first report to multilaterally investigate the relationship between LUTS, the DCSA on MRI, blood data, and lumbar spinal symptoms in a community-dwelling population.

Previous studies have reported LUTS’ prevalence to be 46–80% in patients with LSS [2, 8, 15, 16]. Conversely, the overall prevalence of LUTS in the United States was reported to be 18.7%, increasing with age, which did not differ by sex and race [5]. In Japan, the prevalence of LUTS was reported to be 11.8% in a cross-sectional study [17], and the prevalence in our study was almost equal to that of previous studies targeting all healthy generations. The most frequent stenotic levels were L4/5 and L3/4 in the natural history cohort and surgical LSS cases [18, 19]; however, there were no such trends in this study.

The cutoff size of the mDCSA in OAB was 69 mm^2^ in our study, and mDCSA <70 mm^2^, age, and LBP VAS scores were significantly correlated with OAB in the final stepwise multiple logistic regression analyses. In the final ROC analysis, the cutoff value of the scoring system was 7.5 points; thus, OAB should be suspected when the participant’s mDCSA is <70 mm^2^, they are older than 50 years, and they have an LBP VAS score >25. The DCSA has been reported to be related to the severity of LSS in previous studies. In patients with severe LSS and a DCSA<50 mm^2^, the clinical course may deteriorate with conservative treatment; surgical treatment should thus be selected during the early stage [20, 21].

In addition, patients with a DCSA<75 mm^2^ showed greater postoperative clinical improvement than patients with less stenosis [22]. However, the association between LUTS and radiological findings is still debatable regarding patients with LSS. One study found that patients with LSS had a shorter interpedicular distance and more frequent incidence of a block on myelography in the neuropathic bladder (NB)+ group than in the NB- group [11]. Some studies have indicated a correlation between the dural sac’s APD and LUTS, showing that in patients with LSS and lumbar disc herniation, a dural sac with an APD below 5–8 mm (using CT, CT myelography, or MRI) is an important predictor of NB [8, 10]. There are reports that LUTS and Intermittent claudication both do [9, 23] and do not [10, 24] correlate with the DCSA. In this study, the OABSS was significantly higher in the 70 mm^2^<mDCSA group than in the other groups. In addition, the DCSA was considered useful for detecting LUTS in LSS when assessed with age and LBP; however, further studies are needed to determine which radiological findings are the best for evaluating LSS.

Vascular risk factors such as HT, DL, and DM, play a role in the development of LUTS in both sexes [7]. In this study, patient histories including HT, SBP, and levels of TG, HbA1c, and FGS showed significant between-group differences; however, none of these were independent risk factors. One study reported a decreased association of urine cross-linked NTX with lumbar osteophytes on radiography in Caucasians [25]; however, the K-L grade and bone metabolism markers were not related to OAB in this study.

This study had several limitations. First, the OABSS can evaluate storage but not voiding symptoms; however, LUTS in patients with LSS represent both storage and voiding symptoms [4]. Therefore, we could not study the relationship between the DCSA and voiding symptoms. However, both can be evaluated if the International Prostate Symptom Score [26] is evaluated. Second, LUTS was diagnosed using the OABSS, a self-administered, self-reported history questionnaire; as our study was based on community-dwelling individuals, invasive urological examinations such as urodynamic studies or cystometry could not be performed because of ethical concerns, and we could not directly evaluate the urinary tract’s function. Third, this was a cross-sectional study involving participants from only one area of Japan. Thus, our results may not represent the Japanese population as a whole, and there is a need to evaluate participants with DCSA<70 mm^2^ in a longitudinal study. Finally, the scoring system used in this study implies that OAB should be suspected when the patient is older than 60 years with an LBP VAS score >50, even if the patient’s mDCSA is >70 mm. In this case, OAB might be caused by the urinary tract itself, requiring urological screening. Nevertheless, to the best of our knowledge, this is the first study to have investigated the relationship between DCSA and LUTS in a Japanese population. A longitudinal study is needed to define the relationships between LUTS and DCSA and elucidate the issue of when to intervene.

## Conclusions

The prevalence of LUTS is 11% among community-dwelling individuals in Japan, and the most frequent mDCSA levels in participants with OAB were L3/4 and L4/5. LUTS should be considered a cause of LSS when the mDCSA is <69 mm^2^. Assessing mDCSA with age and the LBP VAS score was more valuable in detecting LUTS in LSS than the mDCSA alone.
